# Supplementary material for: miRNome and Proteome Profiling of Human Keratinocytes and Adipose Derived Stem Cells Proposed miRNA-Mediated Regulations of Epidermal Growth Factor and Interleukin 1-Alpha
Source: Int J Mol Sci. 2023 Mar 4;24(5):4956. doi: 10.3390/ijms24054956 (PMC10002856; doi:10.3390/ijms24054956)
Supplement: Supplementary file 1 [file ijms-24-04956-s001.zip › captions.pdf]

Figure S1. (**a**) Boxplot shows the signal strength of the quality control analysis for each sample involved in the microarray analysis. (**b**) Principal Component Analysis (PCA) reveals the distribution of samples after the quality check. The blue and red colors show the ADSCs and keratinocytes respectively;.

Table S1. Oligonucleotides primers used in qPCR [38,45,83–86].

Table S2. miRNA PCR assays list.

Table S3. List of primary antibodies used in ICC.

Table S4. Microarray sample Quality Check.

Table S5. List of differentially expressed miRNAs.

Table S6. List of upregulated miRNAs and their target genes using miRwalk database.

Table S7. List of downregulated miRNAs and their target genes using miRwalk database.

Table S8. List upregulated miRNAs-targeted genes expressed in skin tissues.

Table S9. Pathway enrichment analysis using the Reactome database for upregulated miRNAs-targeted genes expressed in skin tissues.

Table S10. Proteome profiler array analysis.

Table S11. Predicted miRNA-mediated gene and protein regulations (Integrated analysis).
