# Supplementary material for: miRNome and Proteome Profiling of Human Keratinocytes and Adipose Derived Stem Cells Proposed miRNA-Mediated Regulations of Epidermal Growth Factor and Interleukin 1-Alpha
Source: Int J Mol Sci. 2023 Mar 4;24(5):4956. doi: 10.3390/ijms24054956 (PMC10002856; doi:10.3390/ijms24054956)
Supplement: Supplementary file 1 [file ijms-24-04956-s001.zip › Supplementary Table S1_Proof.pdf]

Supplementary Table S1. Oligonucleotides primers used in the study.

Name	Forward Primer	Reverse Primer	Accession#	Reference
GAPDH qPCR	CCTGCACCACCAACTGCTTA	GGCCATCCACAGTCTTCTGAG	NM_001357943.2	[83]
KRT1	GTTCCAGCGTGAGGTTTGTT	TAAGGCTGGGACAAATCGAC	NM_006121.4	[84]
KRT5	CAGAGCCACCTTCTGCGTCCTG	GCTGAAGCTACGACTGCCC	NM_000424.4	[85]
KRT10	CCATCGATGACCTTAAAAATCAG	GCAGAGCTACCTCATTCTCATACTT	NM_000421.5	[86]
KRT14	CCTCCTCCAGCCGCCAAATCC	TTGGTGCGAAGGACCTGCTCG	NM_000526.5	[85]
KRT18	AGCAAAATCCGGGAGCACTT	GAGCCCATGGATGTCGTTCT	NM_199187.2	Self-designed
p63	GAAAACAATGCCCAGACTCAA	TGCGCGTGGTCTGTGTTA	NM_001329149.2	[87]
U6 snRNA	ATTGGAACGATACAGAGAAGATT	GGAACGCTTCACGAATTTG	-	[88]

Abbreviations: GAPDH (Glyceraldehyde 3-phosphate Dehydrogenase), KRT1 (Keratin 1), KRT5 (Keratin 5), KRT10 (Keratin 10), KRT14 (Keratin 14), KRT18 (Keratin 18), p63 (tumor protein p63).
