# Supplementary material for: miRNome and Proteome Profiling of Human Keratinocytes and Adipose Derived Stem Cells Proposed miRNA-Mediated Regulations of Epidermal Growth Factor and Interleukin 1-Alpha
Source: Int J Mol Sci. 2023 Mar 4;24(5):4956. doi: 10.3390/ijms24054956 (PMC10002856; doi:10.3390/ijms24054956)
Supplement: Supplementary file 1 [file ijms-24-04956-s001.zip › Supplementary Table S2.pdf]

Supplementary Table S2. MiRNA PCR assays used in the study (Qiagen, Germany).

miscript assays	GeneGlobe ID
hsa-miR-30b-5p	MS00003276
hsa-mir-30c-5p	MS00009366
hsa-miR-195-5p	MS00003703
hsa-miR-374a-5p	MS00009604
miRCURY® LNA assays	
U6 snRNA	YP02119464
hsa-miR-29b-3p	YP00204679
hsa-miR-34a-3p	YP00206061
hsa-miR-203a	YP00205914
