# Supplementary material for: miRNome and Proteome Profiling of Human Keratinocytes and Adipose Derived Stem Cells Proposed miRNA-Mediated Regulations of Epidermal Growth Factor and Interleukin 1-Alpha
Source: Int J Mol Sci. 2023 Mar 4;24(5):4956. doi: 10.3390/ijms24054956 (PMC10002856; doi:10.3390/ijms24054956)
Supplement: Supplementary file 1 [file ijms-24-04956-s001.zip › Supplementary Table S3.pdf]

Supplementary Table S3. Primary antibodies used in the study.

Antibody name	Host species	Clone#	Cat#	Working Conc.
**Anti-Cytokeratin 5**	Rabbit	EP1601Y	ab52635	1/100
**Anti-Cytokeratin 14**	Mouse	LL002	ab7800	1/200
**Anti-Cytokeratin 18**	Mouse	C-04	ab668	1/100
