# Supplementary figures and images for: miRNome and Proteome Profiling of Human Keratinocytes and Adipose Derived Stem Cells Proposed miRNA-Mediated Regulations of Epidermal Growth Factor and Interleukin 1-Alpha
Source: Int J Mol Sci. 2023 Mar 4;24(5):4956. doi: 10.3390/ijms24054956 (PMC10002856; doi:10.3390/ijms24054956)

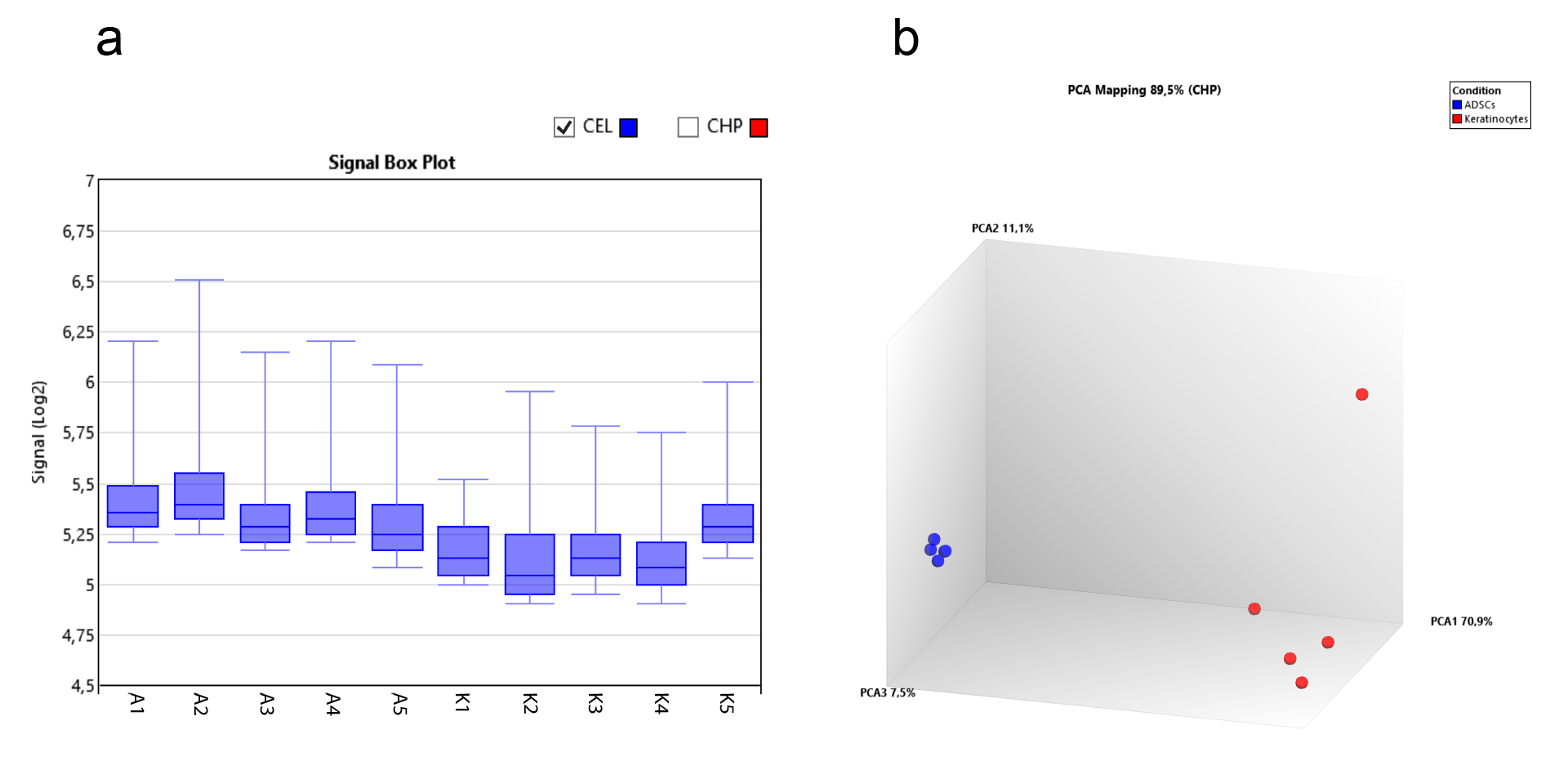

Supplement: Supplementary file 1 [file ijms-24-04956-s001.zip › Supplementary Figure S1-01.png]
